# Supplementary material for: Pressure-tuned quantum criticality in the large-D antiferromagnet DTN
Source: Nat Commun. 2024 Mar 14;15:2295. doi: 10.1038/s41467-024-46527-x (PMC10940708; doi:10.1038/s41467-024-46527-x)
Supplement: Supplementary file 1 — Supplementary Information [file 41467_2024_46527_MOESM1_ESM.pdf]

Supplemental Material for the manuscript "Pressure-tuned quantum criticality in the large-D antiferromagnet DTN"

Kirill Yu. Povarov,[1,*] David E. Graf,[2] Andreas Hauspurg,[1,3] Sergei Zherlitsyn,[1] Joachim Wosnitza,[1,3] Takahiro Sakurai,[4] Hitoshi Ohta,[5,6] Shojiro Kimura,[7] Hiroyuki Nojiri,[7] V. Ovidiu Garlea,[8] Andrey Zheludev,[9] Armando Paduan-Filho,[10] Michael Nicklas,[11] and Sergei A. Zvyagin[1,†]

[1] *Dresden High Magnetic Field Laboratory (HLD-EMFL) and Würzburg-Dresden Cluster of Excellence ct.qmat, Helmholtz-Zentrum Dresden-Rossendorf (HZDR), 01328 Dresden, Germany*
[2] *National High Magnetic Field Laboratory, Tallahassee, Florida 32310, USA*
[3] *Institut für Festkörper- und Materialphysik, Technische Universität Dresden, 01062 Dresden, Germany*
[4] *Research Facility Center for Science and Technology, Kobe University, Kobe, 657-8501, Japan*
[5] *Molecular Photoscience Research Center, Kobe University, Kobe 657-8501, Japan*
[6] *Graduate School of Science, Kobe University, Kobe 657-8501, Japan*
[7] *Institute for Materials Research, Tohoku University, Sendai 980-8578, Japan*
[8] *Neutron Scattering Division, Oak Ridge National Laboratory, Oak Ridge, TN 37831, USA*
[9] *Laboratory for Solid State Physics, ETH Zürich, Switzerland*
[10] *Instituto de Fisica, Universidade de São Paulo, 05315-970 São Paulo, Brazil*
[11] *Max Planck Institute for Chemical Physics of Solids, 01187 Dresden, Germany*
(Dated: February 23, 2024)

This Supplemental Material contains crystallographic information, details of neutron-diffraction experiments under pressure, estimates of lattice compressibility, the numerical/analytical ansatz used to describe the first critical field, various fit procedures, and additional high-pressure ESR measurements.

CONTENTS

I. Crystallographic information 1

II. High-pressure neutron-diffraction experiments 2

III. Fits to extract the critical fields 2

IV. Estimating the spin gap magnitude 2
- A. DMRG results 2
- B. Critical coupling from RPA 4
- C. The gap in three dimensions 4

V. Fits to the experimental phase boundaries 5

VI. Elastic constants 5
- A. Hydrostatic pressure vs uniaxial strain 5
- B. Experimental compressibility 6
- C. Magnetostriction coefficients 6

VII. High-pressure ESR 7
- A. GSWT description 7
- B. ESR frequency-field diagrams 7

References 7

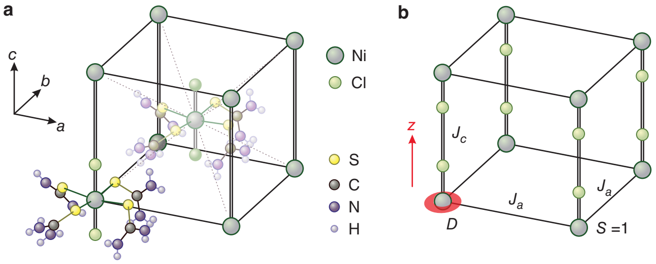

FIG. S1. **a** Crystal structure of DTN, with two $NiCl_2{\cdot}4SC(NH_2)_2$ units per body-centered tetragonal cell. **b** Sketch of the relevant magnetic interactions in DTN. The single-ion easy-plane perpendicular to c (z) direction is highlighted for one of the Ni^{2+} ions.

I. CRYSTALLOGRAPHIC INFORMATION

A schematic view of DTN crystallographic structure (body-centered tetragonal, space group $I4$) is displayed in Fig. S1a. The magnetic properties are determined by the competition between the strong single-ion planar anisotropy D and the antiferromagnetic exchange couplings J_c (along the c direction, Ni-Cl-Cl-Ni superexchange bond) and J_a along the a, b directions. This is illustrated in Fig. S1b. Possible interactions between the two interpenetrating tetragonal sublattices are known to be frustrated and negligible [1].

* k.povarov@hzdr.de; Previous address: Laboratory for Solid State Physics, ETH Zürich, Switzerland
† s.zvyagin@hzdr.de

II. HIGH-PRESSURE NEUTRON-DIFFRACTION EXPERIMENTS

Neutron-diffraction experiments on powder samples were performed using instrument HB2a, at the High Flux Isotope Reactor, Oak Ridge National Laboratory (Tennessee, USA). Up to a few grams of deuterated DTN powder material were used. A Ge[113] or Ge[115] vertically focussing wafer-stack monochromator was used to produce a neutron beam with 2.41 Å or 1.54 Å wavelength. A ^{4}He Orange cryostat with aluminum He-gas pressure cell (capable to produce pressures up to 6 kbar) or a CuBe clamp cell were used.

The pressure in the gas cell was measured *in situ* using a manometer. For the clamp cell a rock salt calibration curve was utilized, that provided accuracy better than 1 kbar.

Selected diffraction datasets (scattered neutron beam intensity vs momentum transfer) are shown in Fig. S2. After the initial data reduction, the `FULLPROF` package [2] was used for the intensity-profile analysis and structure determination. Fit examples are shown in Fig. S3. At small pressures, the peak pattern remains qualitatively the same for all temperatures measured, only the lattice constants are decreasing gradually. The situation changes at around $P_{\mathrm{irr}} \simeq 6$ kbar. Extra Bragg peaks appear, revealing a doubling of the structural unit cell. These peaks are not vanishing upon releasing the pressure (Fig. S2b), evidencing the irreversible character of the phase transition. A more detailed analysis of this transition and the high-pressure structure is given in Ref. [3].

III. FITS TO EXTRACT THE CRITICAL FIELDS

Here, we discuss the fits describing the tunnel-diode-oscillator (TDO) frequency-shift data (Fig. 3 of the main text). For the low-field step-like anomaly, we use the following empirical fit function:

$$\frac{\Delta f}{f} = \left[\frac{\Delta f}{f}\right]_1 + \eta_1 H + \frac{A_1}{2}\left\{1 + \mathrm{erf}\left(\frac{H - H_{c1}}{\delta H_1\sqrt{2}}\right)\right\}. \tag{S.1}$$

Here, the first two terms describe the linear background, and the step-like part is described by the Gaussian error function centered at the first critical field H_{c1}. The parameter δH_1 controls the transition width.

Describing the TDO data at H_{c2} is more complicated, as one has to deal with an asymmetric peak there. We use the following empirical formula:

$$\frac{\Delta f}{f} = \left[\frac{\Delta f}{f}\right]_2 + \eta_2 H + \begin{cases} \dfrac{A_2}{\delta H_2\sqrt{2\pi}}\exp\left[-\dfrac{1}{2}\left(\dfrac{H - H_{c2}}{\delta H_2}\right)^2\right], & H \geq H_{c2} \\ B + \dfrac{A'}{(H_\infty - H)^\alpha}, & H < H_{c2} \end{cases}. \tag{S.2}$$

Here, the high-field part of the peak is described by a Gaussian distribution of weight A_2 and width δH_2, centered at H_{c2}. Below this critical field, the broad part is better captured by a generalized hyperbola, centered at some effective field $H_\infty > H_{c2}$, with the amplitude A' and the characteristic exponent α. The offset $B = \frac{A_2}{\delta H_2\sqrt{2\pi}} - \frac{A'}{(H_\infty - H_{c2})^\alpha}$ ensures that the curve is continuous at H_{c2}. The linear background, characterized by $\left[\frac{\Delta f}{f}\right]_2$ and η_2, is the same for both parts of the curve. This background is subtracted from the full range of the data in order to yield the detrended version of the plots actually shown in Fig. 3.

IV. ESTIMATING THE SPIN GAP MAGNITUDE

A. DMRG results

As mentioned in the main text, we simulated an $N = 249$ sites $S = 1$ chain, using the density matrix renormalization group (DMRG) implementation in the `Julia` version of `ITensors` package (30 sweeps, energy convergence better than 10^{-6}). In particular, it allows for a straightforward gap-size estimation. First, one finds the ground-state vector in the Hilbert space of the model, which is a conventional DMRG routine. Then, this ground state is excluded from the Hilbert space, and the DMRG procedure is repeated once again. Naturally, the lowest energy state yielded is then the first excited state. The energy difference between the two respective states corresponds to the energy gap Δ_0. The calculation results are shown in Fig. S4. They provide a very good interpolation between the two known limits of the model. On the large-D end the generalized spin-wave

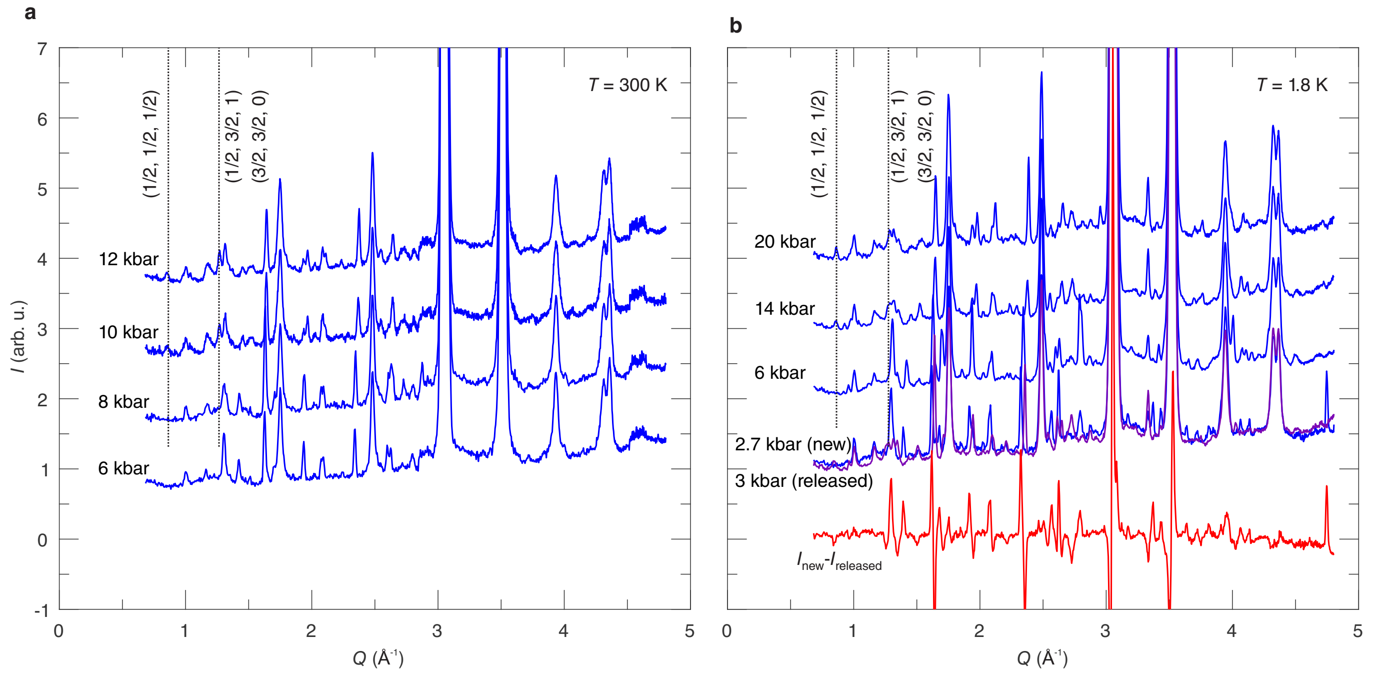

FIG. S2. Measured neutron-diffraction intensity vs momentum transfer at different pressures [3]. **a** Data at 300 K, clamp cell. The data is offset for clarity. The dotted lines indicate the position of some Bragg peaks that correspond to the lattice period doubling. **b** Data at 1.8 K, clamp cell. The 3 kbar curve obtained after the pressure release (purple), is overlayed with the 2.7 kbar curve obtained at the initial loading cycle (blue). The intensity difference between these two measurements is shown below in red.

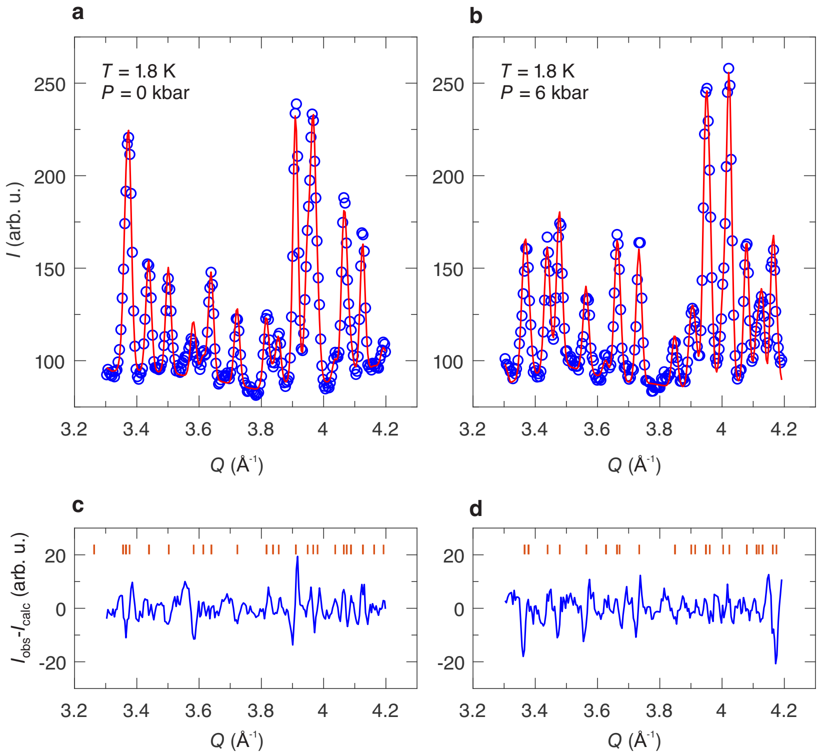

FIG. S3. Examples of fits (red lines) to the powder diffraction data (open circles) of DTN [3]. **a** Data at ambient pressure. **b** Data at 6 kbar. **c,d** The residues and the expected Bragg peak positions.

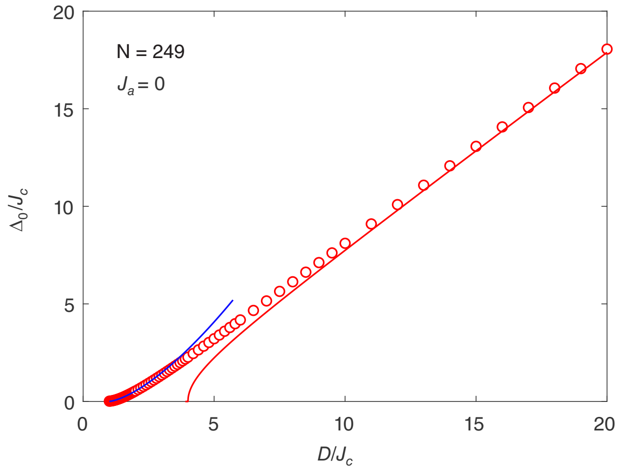

FIG. S4. The gap Δ_0/J_c in the disordered large-D phase for the one-dimensional model as function of D/J_c. The points are the results of DMRG calculations. The red solid line represents GSWT results in the large-D limit. The blue line shows the critical gap scaling near $D/J_c \simeq 1$.

theory (GSWT) is applicable. It describes the spin-gap as $\sqrt{D^2 - 4DJ_c}/J_c$ [4–6]. On the other end, in the vicinity of the critical point one finds the spin-gap critical behavior $0.52(D/J_c - 0.971)^{1.478}$ [7]. These two regimes are shown as red and blue lines in Fig. S4 correspondingly.

B. Critical coupling from RPA

Here, we give a detailed description of the random phase approximation (RPA)-based *ansatz*, which we used to calculate the gap (and, hence, H_{c1}) as a function of pressure. The starting point of our calculation is the RPA (or a mean-field, if one prefers such notation) expression for the transverse staggered susceptibility [8]:

$$\chi_{xx}^{\prime\mathrm{RPA}}(\mathbf{Q}) = \frac{\chi_{xx}^{\prime 1\mathrm{D}}(\pi/c)}{1 - 4J_a \chi_{xx}^{\prime 1\mathrm{D}}(\pi/c)}. \tag{S.3}$$

Here, the three-dimensional antiferromagnetic propagation vector is $\mathbf{Q} = (\pi/a,\ \pi/a,\ \pi/c)$, and $\chi_{xx}^{\prime 1\mathrm{D}}(\pi/c)$ is the transverse staggered susceptibility of a single chain. Eq. (S.3) describes the critical susceptibility that diverges once we enter the domain of planar long-range order.

To evaluate this quantity, we employ certain dynamic structure-factor approximations for large D and the Kramers–Kronig relations. The useful RPA-type approximation we make for the transverse dynamic structure factor in a large-D chain of N sites is:

$$\mathcal{S}^{xx}(\pi/c, \omega) = N \frac{\mathcal{A}D}{\Delta_0} \delta(\Delta_0 - \omega). \tag{S.4}$$

The dispersion relation has a minimum at the wavevector π/c. The excitation energy at this minimum is the gap Δ_0. The amplitude prefactor $\mathcal{A}$ is, generally, a complex quantity that depends on D and J_c, as well as on the on-site and inter-site ground-state correlators $\left\langle (\hat{S}^x)^2 \right\rangle$ and $\left\langle \hat{S}_n^\alpha \hat{S}_{n+1}^\alpha \right\rangle$, with $\alpha = x, z$ [9, 10]. However, in first approximation this is merely $\mathcal{A} = 1 + \mathcal{O}(J_c/D)$. Treating $\mathcal{A}$ as a constant somewhat larger than 1 is the approximation which we adopted in our *ansatz* beyond conventional RPA.

Then, at $T = 0$ the dissipative part of the transverse staggered susceptibility has the following form according to the fluctuation-dissipation theorem:

$$\chi_{xx}''(\pi/c, \omega) = \frac{\pi}{N} \mathcal{S}^{xx}(\pi/c, \omega) = \frac{\pi \mathcal{A} D}{\Delta_0} \delta(\Delta_0 - \omega). \tag{S.5}$$

Now, using the Kramers–Kronig relations, one can evaluate the desired static susceptibility, which is equivalent to the reactive part at $\omega = 0$:

$$\chi_{xx}'(\pi/c, 0) = \frac{1}{\pi} \int_{-\infty}^{+\infty} \frac{\pi \mathcal{A} D}{\Delta_0} \frac{\delta(\Delta_0 - \Omega)}{\Omega} d\Omega \tag{S.6}$$

We now have obtained the following estimate for the one-dimensional critical susceptibility:

$$\chi_{xx}'(\pi/c) = \frac{\mathcal{A}D}{\Delta_0^2}, \tag{S.7}$$

which, combined with the RPA expression (S.3) results in the ordering criterion:

$$4J_a \frac{\mathcal{A}D}{\Delta_0^2} = 1. \tag{S.8}$$

This is the criterion (5) from the main text.

C. The gap in three dimensions

Now, we can estimate the influence of J_a bonds on the spin gap Δ_0. This gap value, modified by the three-dimensional interactions, we label $\Delta_{3\mathrm{D}}$. Using this notation, we can write a three-dimensional analogue of Eq. (S.7):

$$\chi_{xx}^{\prime\mathrm{RPA}}(\mathbf{Q}) = \frac{\mathcal{A}D}{\Delta_{3\mathrm{D}}^2}, \tag{S.9}$$

where we assume a constant factor $\mathcal{A}$. Then, using Eqs. (S.3) and (S.7), we obtain

$$\Delta_{3\mathrm{D}}^2 = \Delta_0^2 - 4\mathcal{A}DJ_a. \tag{S.10}$$

Using the critical coupling $J_a^{\rm crit}$ as defined for given D and J_c by (S.8), we can rewrite this result in an alternative form:

$$\Delta_{\rm 3D}^2 = \Delta_0^2 - \Delta_0^2 \frac{J_a}{J_a^{\rm crit}}. \qquad (S.11)$$

This is the Eq. (6) from the main text.

V. FITS TO THE EXPERIMENTAL PHASE BOUNDARIES

A given set of DTN spin-Hamiltonian parameters can be represented by a point in the Sakai–Takahashi phase diagram plane (Fig. 7(d) of the main text, where we denote such points by the purple open circles) [11]. The Hamiltonian parameters D and J_c are changing with pressure, so the corresponding point on the diagram also changes its position. Since the change in D and J_c is linear, and J_a is a constant, the resulting trajectory in this “phase space” is nearly a straight line. Such line may have only a single interception with the curved phase boundary between the gapped and long-range ordered states. The slope of the line that represents the trajectory is given by the ratio between $\partial D/\partial P$ and $\partial J_c/\partial P$. Since the experimentally known pressure dependence of H_{c2} locks the linear relationship between these quantities, finding this slope is effectively a single-parameter problem. The critical pressure P_c is then ideally suited to play the role of this open parameter. The parameterized trajectory we are discussing is crossing the known phase boundary at $P = P_c$, which allows the evaluation of the corresponding $\partial D/\partial P$ and $\partial J_c/\partial P$ values. Pairs of parameters, obtained for different choices of P_c, are shown in Fig. S5b in the magnetostriction notation (see the next section). Since the value of P_c remains *the only parameter* in the description of the experimental data, we can utilize a numeric criterion to optimize the agreement. As such criterion, we consider the mean-square deviation

$$\chi^2 = \mu_0 \sqrt{\sum \left(H_{c1}^{\rm obs} - H_{c1}^{\rm calc}\right)^2}, \qquad (S.12)$$

with the calculated H_{c1} values obtained from our *ansatz*, and $H_{c1}^{\rm obs}$ being the experimental data. This quantity is shown in Fig S5a. The deviation is robustly minimized in the range $P_c = 4.2 \pm 0.3$ kbar, in very good agreement with the “apparent” value of the critical pressure.

VI. ELASTIC CONSTANTS

The pressure dependencies of D and J_c we have found can be expressed as magnetostriction coefficients. However, under a hydrostatic-pressure conditions the relation between the length reduction and the Young's modulus for the given direction is not straightforward. Here we derive the corresponding equations prior to discussing the measured lattice-parameters changes under pressure.

A. Hydrostatic pressure vs uniaxial strain

We need to consider the elasticity-theory equations for three different cases: hydrostatic pressure, uniaxial strain along the x direction, and uniaxial strain along z. Since we are dealing with a tetragonal crystal, we can neglect the shear components. Than, the equations that define the relation between the stress σ and the strain ε are reduced to a 3-by-3 matrix equation:

$$\begin{pmatrix}\sigma_x\\ \sigma_y\\ \sigma_z\end{pmatrix} = \mathbb{C}\begin{pmatrix}\varepsilon_x\\ \varepsilon_y\\ \varepsilon_z\end{pmatrix}. \qquad (S.13)$$

Here, the elasticity matrix $\mathbb{C}$ is composed of several symmetry-allowed elastic moduli:

$$\mathbb{C} = \begin{pmatrix} c_{xx} & c_{xy} & c_{xz}\\ c_{xy} & c_{xx} & c_{xz}\\ c_{xz} & c_{xz} & c_{zz}\end{pmatrix}. \qquad (S.14)$$

The inverse problem $\vec{\varepsilon} = \mathbb{S}\vec{\sigma}$ is formulated with the help of the compliance matrix $\mathbb{S} = \mathbb{C}^{-1}$, which has the same symmetry. The compliance-matrix components are [12]:

$$\begin{aligned} s_{xx} &= \frac{c_{xx}c_{zz} - c_{xz}^2}{c_0(c_{xx} - c_{xy})},\\ s_{zz} &= \frac{c_{xx} + c_{xy}}{c_0},\\ s_{xy} &= \frac{c_{xz}^2 - c_{xy}c_{zz}}{c_0(c_{xx} - c_{xy})},\\ s_{xz} &= -\frac{c_{xz}}{c_0},\\ &\text{with } c_0 = c_{zz}(c_{xx} + c_{xy}) - 2c_{xz}^2. \end{aligned} \qquad (S.15)$$

For a uniaxial stress applied along the x direction, $\vec{\sigma} = (P,\ 0,\ 0)$. Thus, the strain along that direction is:

$$\varepsilon_x = s_{xx}P.$$

For the z direction, with $\vec{\sigma} = (0,\ 0,\ P)$ we correspondingly have

$$\varepsilon_z = s_{zz}P.$$

This gives us the conventional Young's moduli

$$E_{xx} = s_{xx}^{-1} = c_{xx} - c_{xy} + \frac{(c_{zz}c_{xy} + c_{xz}^2)(c_{xx} - c_{xy})}{c_{xx}c_{zz} - c_{xz}^2}, \qquad (S.16)$$

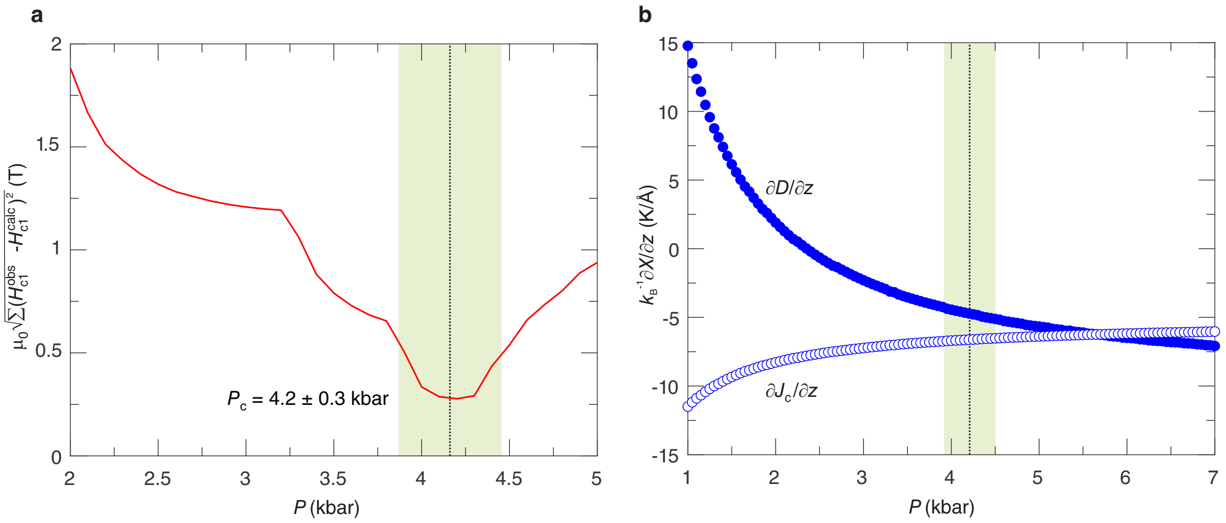

FIG. S5. The model results depending on the selected critical pressure value $P = P_c$. **a** Mean-square deviation between the RPA *ansatz* fit and the actually observed values of H_{c1} as a function of pressure that we assign as critical. The agreement is optimized around $P_c \simeq 4.2$ kbar; the error bar represents the approximate threshold at which the χ^2 (see Eq. (S.12)) doubles compared to its minimal value. **b** The magnetostriction coefficients as a function of pressure that we assign as critical. The optimal region is highlighted.

and

$$E_{zz} = s_{zz}^{-1} = c_{zz} - 2\frac{c_{xz}^2}{c_{xx}+c_{xy}}. \tag{S.17}$$

Under hydrostatic pressure the compression is different. The stress is $\vec{\sigma} = (P,\ P,\ P)$, and the strain is $\vec{\varepsilon} = (\varepsilon_x,\ \varepsilon_x,\ \varepsilon_z)$. For the strain components, we find:

$$\varepsilon_x = (s_{xx}+s_{xy}+s_{xz})P = \frac{c_{zz}-c_{xz}}{c_{zz}(c_{xx}+c_{xy})-2c_{xz}^2}P,$$
$$\varepsilon_z = (2s_{xz}+s_{zz})P = \frac{c_{xx}+c_{xy}-2c_{xz}}{c_{zz}(c_{xx}+c_{xy})-2c_{xz}^2}P. \tag{S.18}$$

Hence, under the hydrostatic conditions our pressure-to-strain ratios are:

$$\left[\frac{\Delta P}{\Delta a/a}\right] = \frac{c_{zz}(c_{xx}+c_{xy})-2c_{xz}^2}{c_{zz}-c_{xz}}, \tag{S.19}$$

$$\left[\frac{\Delta P}{\Delta c/c}\right] = \frac{c_{zz}(c_{xx}+c_{xy})-2c_{xz}^2}{c_{xx}+c_{xy}-2c_{xz}}. \tag{S.20}$$

Note, that from Eqs. (S.17) and (S.20) follows $\left[\frac{\Delta P}{\Delta c/c}\right] = E_{zz}\frac{c_{xx}+c_{xy}}{c_{xx}+c_{xy}-2c_{xz}}$. We obtain a significant boost in the compression compared to the uniaxial case due to off-diagonal components of the elasticity matrix.

B. Experimental compressibility

As demonstrated above, the hydrostatic compressibility modulus for the c direction is:

$$\left[\frac{\Delta P}{\Delta c/c}\right] = E_{zz}\frac{c_{xx}+c_{xy}}{c_{xx}+c_{xy}-2c_{xz}}. \tag{S.21}$$

Here, E_{zz} is the true Young's modulus for longitudinal stress, and $c_{\alpha\beta}$ are the various elastic moduli. According to Ref. [13], these moduli are $c_{xx} = 26.1$ GPa, $c_{xy} = 15.3$ GPa, and $c_{xz} = 12.4$ GPa, yielding a factor 4.76 in Eq. (S.21). We find $\left[\frac{\Delta P}{\Delta c/c}\right] = 23.1 \pm 1.7$ GPa, hence $E_{zz} = 4.9 \pm 0.4$ GPa. This is reasonably close to the value 7.5 ± 0.7 GPa [13]. Possible systematic errors in the determination of the elastic moduli $c_{\alpha\beta}$ would affect either estimate.

C. Magnetostriction coefficients

Using the measured lattice parameter dependence and derived $\partial D/\partial P$, $\partial J_c/\partial P$, we can estimate the magnetostriction coefficients. Let z parameterize the bond length along the c direction, then:

$$\frac{\partial X}{\partial z} = \frac{\partial X}{\partial P}\left(\frac{\partial c}{\partial P}\right)^{-1}.$$

Within this model, the magnetostriction coefficients $k_B^{-1}\partial J_c/\partial z$ and $k_B^{-1}\partial D/\partial z$ as function of critical pressure choice are shown in Fig. S5b. The possible values within the optimal P_c range are highlighted; $k_B^{-1}\partial D/\partial z = -4.1 \pm 0.7$ K/Å and $k_B^{-1}\partial J_c/\partial z = -6.5 \pm 0.3$ K/Å. Surprisingly, they noticeably deviate from the set of magnetostriction coefficients (0 and -2.5 K/Å) previously

found at the zero pressure [13]. The latter values are clearly insufficient to describe the rapid growth of H_{c2} under pressure, yielding only 0.25 T/kbar (which is about 1/3 of the actual slope).

VII. HIGH-PRESSURE ESR

A. GSWT description

The key idea of generalized spin wave theory (GSWT) [6, 14] is to approximate the collective ground state of a magnetic system as a simple product of single-ion states. Both, quantum-disordered states (products of local singlets $|0\rangle_i$) and magnetic states with $\left\langle \hat{\mathbf{S}}_i \right\rangle \neq 0$ (products of general combinations of $|0\rangle_i$,$|-1\rangle_i$, $|+1\rangle_i$) are examples of such states. The particular mixing weights in such combinations can be found from minimizing the mean-field energy. This is one of the crucial differences to the linear spin wave theory (LSWT) where a ground state with $|\left\langle \hat{\mathbf{S}}_i \right\rangle| = S$ is postulated. Within GSWT, arbitrary values of $|\left\langle \hat{\mathbf{S}}_i \right\rangle| \leq S$ are possible, partially accounting for the effect of quantum fluctuations.

Having identified the mean-field ground state on every ion, the remaining two states of the appropriate single-spin basis sets remain as excited states. It is now possible to rewrite the Hamiltonian in the second-quantization notation associating the bosonic particle creation operators $\hat{a}_i^\dagger, \hat{b}_i^\dagger$ with creating these local excited states. This is another difference to LSWT that technically accounts only for the first excited state. By Fourier transformation of the bosonic operators we can obtain the second-quantization Hamiltonian in the momentum space representation. Then it can be diagonalized by Bogolyubov transformation, yielding the dispersion relations for the possible excitation branches. We handle these calculations numerically for the given Hamiltonian parameters using our own code. Then the excitations at $q = 0$ are the ones of interest for ESR experiments.

We would also like to note that the recently released `Sunny` library (for simulating $\mathrm{SU}(N)$ dynamics in systems of complex magnetic ions) [15] utilizes the same approach and produces identical results for the case of DTN.

B. ESR frequency-field diagrams

The method outlined above allows us to calculate the frequency-field dependence of the uniform, $q = 0$ excitations for DTN at different pressures. Three modes (labeled as A, B, and C) are the "collective" descendants of $|0\rangle \to |\mp 1\rangle$ single-ion transitions at low fields, or the $|+1\rangle \to |0\rangle$ transition at high fields.

In Fig. S6, we show the frequency-field diagrams for the uniform magnetic excitations obtained from ESR experiments at all measured pressures. We also include the reference zero-pressure data from Ref. [16]. The solid lines in Fig. S6 represent the predicted zero-temperature ESR single-magnon transitions for the DTN spin Hamiltonian at corresponding pressures.

Qualitatively the weakly changing spectra are consistent with the above analysis (e.g., pressure-induced shift to the right for the branch B, and to the left for the branch A in both theory and experiment). However, the description of the spectrum in the field range, corresponding to the low-T, long-range ordered-phase is not accurate. At zero pressure, the central part of the spectrum is known to show a strong temperature dependence between 2 and 0.5 K [1], eventually matching the theoretical prediction at $T = 0$. The mismatch appears to be result of the simultaneous action of thermal and quantum fluctuations, not fully accounted for by GSWT. The case of DTN under pressure would be an excellent benchmark for the possible future extensions of the theory incorporating such corrections.

[1] S. A. Zvyagin, J. Wosnitza, A. K. Kolezhuk, V. S. Zapf, M. Jaime, A. Paduan-Filho, V. N. Glazkov, S. S. Sosin, and A. I. Smirnov, Spin dynamics of $\mathrm{NiCl_2}$-4SC$(\mathrm{NH_2})_2$ in the field-induced ordered phase, Phys. Rev. B **77**, 092413 (2008).

[2] J. Rodríguez-Carvajal, Recent advances in magnetic structure determination by neutron powder diffraction, Physica B **192**, 55 (1993).

[3] A. Mannig, *Experimental studies of zero-field phase transitions in quantum magnets* (PhD thesis, ETH Zürich, 2017).

[4] N. Papanicolaou and P. Spathis, Quantum spin-1 chains with strong planar anisotropy, J. Phys.: Cond. Mat. **2**, 6575 (1990).

[5] M. Matsumoto and M. Koga, Longitudinal spin-wave mode near quantum critical point due to uniaxial anisotropy, J. Phys. Soc. Jap. **76**, 073709 (2007).

[6] Z. Zhang, K. Wierschem, I. Yap, Y. Kato, C. D. Batista, and P. Sengupta, Phase diagram and magnetic excitations of anisotropic spin-one magnets, Phys. Rev. B **87**, 174405 (2013).

[7] A. F. Albuquerque, C. J. Hamer, and J. Oitmaa, Quantum phase diagram and excitations for the one-dimensional $S = 1$ Heisenberg antiferromagnet with single-ion anisotropy, Phys. Rev. B **79**, 054412 (2009).

[8] J. Jensen and A. R. Mackintosh, *Rare earth magnetism: structures and excitations*, International series of monographs on physics (Clarendon Press, U.K., 1991).

[9] P.-A. Lindgård, Correlation theory of crystal field and anisotropic exchange effects, J. Magn. Magn. Mater. **52**, 47 (1985).

[10] I. Zaliznyak and S. Lee, Magnetic neutron scattering, in *Modern Techniques for Characterizing Magnetic Materials*, edited by Y. Zhu (Springer US, Boston, MA, 2005)

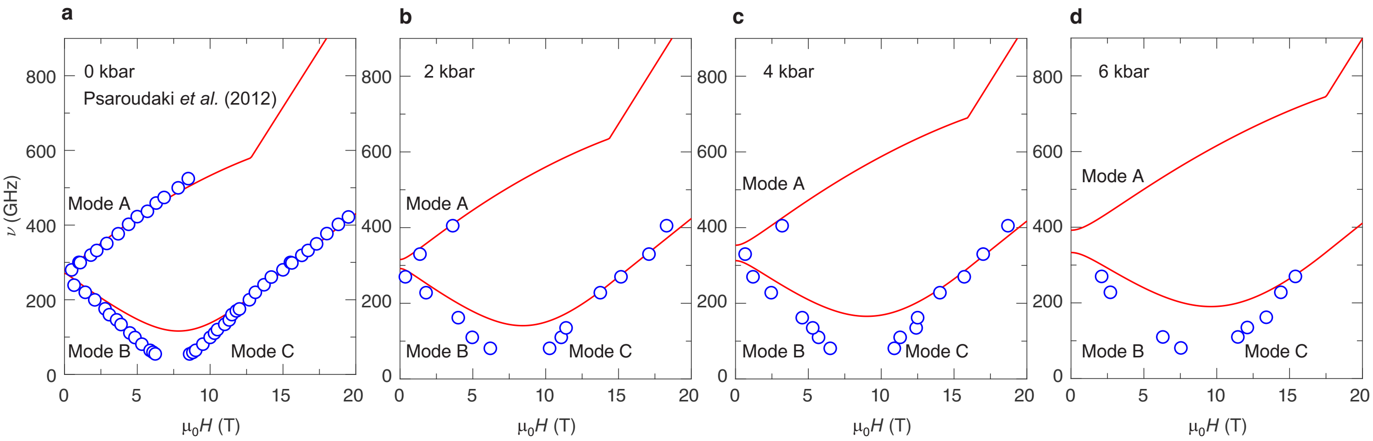

FIG. S6. Frequency–field diagram of the spin excitations in DTN under different pressures. **a** Zero-pressure $T \simeq 2$ K data taken from Ref. [16] (open circles). Solid lines correspond to a $T = 0$ GSWT description of the spin dynamics. **b** Data at $T \simeq 4$ K and 2 kbar pressure (open circles), 2 kbar GSWT calculations (red lines). **c** Same for 4 kbar. **d** Same for 6 kbar.

pp. 3–64.

[11] T. Sakai and M. Takahashi, Effect of the Haldane gap on quasi-one-dimensional systems, Phys. Rev. B **42**, 4537 (1990).

[12] R. F. S. Hearmon, The Elastic Constants of Anisotropic Materials, Rev. Mod. Phys. **18**, 409 (1946).

[13] V. S. Zapf, V. F. Correa, P. Sengupta, C. D. Batista, M. Tsukamoto, N. Kawashima, P. Egan, C. Pantea, A. Migliori, J. B. Betts, M. Jaime, and A. Paduan-Filho, Direct measurement of spin correlations using magnetostriction, Phys. Rev. B **77**, 020404 (2008).

[14] R. A. Muniz, Y. Kato, and C. D. Batista, Generalized spin-wave theory: Application to the bilinear–biquadratic model, Progr. Theor. Exp. Phys **2014**, 1 (2014).

[15] K. Barros, S. Matin, Y. W. Li, M. Wilson, and US-DOE Office of Science, Contributing to Sunny open source code (2022).

[16] C. Psaroudaki, S. A. Zvyagin, J. Krzystek, A. Paduan-Filho, X. Zotos, and N. Papanicolaou, Magnetic excitations in the spin-1 anisotropic antiferromagnet $NiCl_2$-$4SC(NH_2)_2$, Phys. Rev. B **85**, 014412 (2012).
